# Early childhood and adolescent risk factors for psychotic depression in a general population birth cohort sample

**DOI:** 10.1007/s00127-020-01835-7

**Published:** 2020-02-13

**Authors:** Miika Nietola, Hanna Huovinen, Anni Heiskala, Tanja Nordström, Jouko Miettunen, Jyrki Korkeila, Erika Jääskeläinen

**Affiliations:** 1 Psychiatric Department, University of Turku and the Hospital District of Southwest Finland, Kunnallissairaalantie 20, Building 9, 3. Floor, 20700 Turku, Finland; 2 Center for Life Course Health Research, University of Oulu, Oulu, Finland; 3 Medical Research Center Oulu, Oulu University Hospital and University of Oulu, Oulu, Finland; 4 Infrastructure for Population Studies, Faculty of Medicine, University of Oulu, Oulu, Finland; 5 Psychiatric Department, University of Turku and Satakunta Hospital District, Turku, Finland; 6 Department of Psychiatry, Oulu University Hospital, Oulu, Finland

**Keywords:** Psychotic depression, Risk factors, Epidemiology, Birth cohort, Psychosis

## Abstract

**Background and purpose:**

In the group of severe mental disorders, psychotic depression (PD) is essentially under-researched. Knowledge about the risk factors is scarce and this applies especially to early risk factors. Our aim was to study early childhood and adolescent risk factors of PD in a representative birth cohort sample with a follow-up of up to 50 years.

**Methods:**

The study was carried out using the Northern Finland Birth Cohort 1966 (NFBC 1966). We used non-psychotic depression (NPD) (*n* = 746), schizophrenia (SZ) (*n* = 195), psychotic bipolar disorder (PBD) (*n* = 27), other psychoses (PNOS) (*n *= 136) and healthy controls (HC) (*n* = 8200) as comparison groups for PD (*n* = 58). We analysed several potential early risk factors from time of birth until the age of 16 years.

**Results:**

The main finding was that parents’ psychiatric illness [HR 3.59 (1.84–7.04)] was a risk factor and a high sports grade in school was a protective factor [HR 0.29 (0.11–0.73)] for PD also after adjusting for covariates in the multivariate Cox regression model. Parental psychotic illness was an especially strong risk factor for PD. The PD subjects had a parent with psychiatric illness significantly more often (*p* < 0.05) than NPD subjects. Differences between PD and other disorder groups were otherwise small.

**Conclusions:**

A low sports grade in school may be a risk factor for PD. Psychiatric illnesses, especially psychoses, are common in the parents of PD subjects. A surprisingly low number of statistically significant risk factors may have resulted from the size of the PD sample and the underlying heterogeneity of the etiology of PD.

**Electronic supplementary material:**

The online version of this article (10.1007/s00127-020-01835-7) contains supplementary material, which is available to authorized users.

## Introduction

Psychotic depression (PD) is a severe and under-researched disorder with a mostly similar clinical course than psychotic bipolar disorder (PBD), worse than in non-psychotic depression (NPD), but better than in schizophrenia (SZ) [1, 2]. The large diagnostic conversion from PD to other diagnoses and the difficulty of properly identifying patients with PD present challenges to both clinical practice and research, and altogether our understanding of the relationship between PD and other psychotic and depressive illness is far from sufficient [3]. Supporting the validity of a separate diagnostic entity of PD, psychotic symptoms seem to occur with a high probability in recurrent depressive episodes in PD [4].

To understand the etiology of PD, and possible differences in comparison to other affective and psychotic illnesses, we need to know more about the risk factors. Very few studies have been conducted to discover the risk factors of psychotic depression, and especially early life risk factors [2]. There is only one previous Danish register-based study with a follow-up starting from birth. In their study the PD subjects did not differ from NPD or healthy control (HC) subjects regarding birth weight and gestational age. There was a trend in PD that low maternal age and high paternal age served as risk factors [5].

PD patients have been found to suffer more often from childhood traumatic events than NPD patients [6]; however, another study did not find this difference [7]. In the ÆSOP-study in the UK, childhood adversity was associated with PD. The same study found more neurological soft signs in PD patients than in control subjects without a psychotic illness [8].

Concerning other factors, the ethnicity of PD patients has in several studies been more often non-Caucasian [2]. Also, rural residence has been more common among PD patients than NPD patients [9]. Loss of a relative, and especially the loss of the mother, seems to function as risk factor for PD [5].

Parental mental illness is a known risk factor for a variety of different psychiatric disorders [10]. For PD, there are studies showing more family history of mental illness in general [8, 11] and a larger amount of parental mental illness in PD than NPD [5], but there are also contradictory results [12, 13]. There is some evidence that bipolar disorder [5, 14] and psychosis [8, 15] are more common in the families of PD. Family history of depression has not been found to be significantly more common in PD vs. NPD, possibly due to small sample sizes [16–18].

Altogether, the PD risk factor profile has been shown to overlap highly with NPD [5], and to have many more similarities with SZ than with PBD [8]. This conflicts with outcome studies that posit PD as near PBD and separate it from SZ and NPD. Most studies to date have utilized cross-sectional, retrospectively collected information and early risk factors are mostly unknown.

### Aims of the study

Our aim in this study was to analyse several early childhood and adolescent risk factors of PD in the prospectively collected, general population based Northern Finland Birth Cohort 1966 (NFBC 1966). One purpose was to discover potential differences and similarities in risk factor profiles between disorder groups, comparing PD to NPD, SZ, PBD, PNOS and HC.

## Methods

### Sample

Our data came from the NFBC 1966 [19]. The cohort utilizes nationwide registers of hospitalization and outpatient care in Finland in addition to questionnaires and clinical examination data. Psychiatric diagnoses of the NFBC 1966 members were gained from several national registers: Care Register for Health Care was used to acquire diagnoses of all general and psychiatric hospitalizations from birth until the end of 2016. Outpatient diagnoses came from the Finnish outpatient register (specialized care 1998–2016; primary care 2011–2016). Register information about drug reimbursement (until 2005), sick days (until 1999) and disability pensions (until 2015), were also used to gather the lifetime diagnoses of individuals in the cohort.

All the study groups were formed according to lifetime diagnosis, which was defined by the hierarchically highest diagnosis in the data. The hierarchical order starting from the top was SZ (*n* = 195), PBD (*n* = 27), PD (*n* = 58), PNOS (*n* = 136), NPD (*n* = 746), and HC (*n* = 8200). The hierarchical system is described in more detail elsewhere [20]. The diagnoses of each study group are presented in Table 1. In the PD group, all lifetime diagnoses were manually checked. In a previous study of the cohort by our group [20], there were 55 PD subjects until the end of 2013, 2 of whom were converted to schizophrenia or bipolar disorder afterwards. There were also five new PD cases, resulting in the current PD sample size of 58. The HC group subjects did not have any lifetime psychiatric diagnoses, which means we excluded subjects with disorders other than those of the comparison groups. Also, cohort participants who died or moved to another country before the age of 16 years were excluded.

**Table 1:** Diagnostic categories based on ICD 8–10 used in the current study

	ICD-8	ICD-9	ICD-10
Psychotic depression (PD)	2960, 2980	2961E	F32.3, F33.3
Non-psychotic depression (NPD)	3004, 7902	3004	F32.0-F32.2, F32.8-F33.2, F33.4-F33.9, F34.1, F38.10
Schizophrenia (SZ)	295, 2954, 2957	295, 2954, 2957	F20, F25
Psychotic bipolar disorder (PBD)	2961–2969	2962E, 2963E, 2964E, 2967	F30.2, F31.2, F31.5
Other psychoses (PNOS)	297, 298 (except 2980), 299	297, 2988, 2989	F22, F23, F24, F28, F29
Healthy controls (HC)	No psychiatric diagnoses	No psychiatric diagnoses	No psychiatric diagnoses

### Risk factors

Since there is very little research on the risk factors of PD, we performed an explorative analysis of risk factors that have been studied in earlier studies of SZ, PBD or NPD. The risk factors were grouped into the following categories: *psychiatric and somatic illness of the parents, psychosocial risk factors, biological risk factors, school performance and risk of psychotic depression and other mental disorders* and *health-related risk factors at age 14 years.*

#### Psychiatric and somatic illness of the parents

Data concerning the *psychiatric and somatic illness of the parents* was gathered from the same registers, listed above, as the study subjects. Regarding somatic illness, we analysed long hospitalizations (≥ 30 days) of both parents separately as potential risk factors [21]. Only hospitalizations that took place before the end of 1982, i.e. the year when the study subject reached the age of 16 years, were taken into account. We chose this variable because separation from and possible fear of loss of the parent represents a traumatic event for the child. Childhood traumatic events are known to be associated with many psychiatric disorders later in life [22]. Psychiatric illness (*any psychiatric illness, psychosis, schizophrenia, depression, bipolar disorder* and *alcohol use disorder*) of the parents before the study subject reached the age of 16 years was analysed using the same register information as with study group subjects.

#### Psychosocial risk factors

We divided psychosocial risk factors into *risk factors during birth* and *risk factors at age 14 years*. The same questionnaire from 1965 to 1966 for biological risk factors was used for the *risk factors during birth*: *urbanicity, mother’s education, social class in 1966 (determined by father’s occupation), mother’s antenatal mood* [23] and *unwantedness of pregnancy *[24]. In 1980, at the age of 14 years, the cohort members filled out a questionnaire, which provided information about the subjects’ *family type, social class* in 1980 (determined by father’s occupation), *moving hometown in 1966–1980* and *mother’s work status.* In the questionnaire, participants were also asked whether one or several of their siblings were previously deceased and to write down the number. The *deceased siblings* variable is based on this question and has two categories: yes/no. This variable is also an indicator of a traumatic childhood event (see above for *psychiatric and somatic illness of the parents*).

#### Biological risk factors

Information about *birth weight, gestational age, birth weight/gestational age, parity, paternal and maternal age, mother’s antenatal smoking *[23] and *perinatal complications* [25] were collected in a questionnaire that was filled in by an interviewing nurse at the antenatal clinic and at birth in 1965–1966.

Motor development of all children in Finland is regularly followed by nurses and doctors in Finnish welfare clinics as a routine procedure; the child is observed and the parents interviewed. The age when child achieves a particular milestone is written on a welfare card and we used the information on these cards about motor development. We selected the *ages when the child learns to walk and stand without support* as indicators of early motor development [21].

#### School performance and risk of psychotic depression and other mental disorders

The register information from the National Board of Education was combined with the postal questionnaire from 1980 to confirm the *school level of the persons at the age of 14 years.* Data about the study subjects’ *school grades* at the age of 16 years were obtained in 1982 from the Finnish national application system for upper secondary education register. The National Board of Education defines the criteria for school grades and they are same in all Finnish schools [26]. Pupils were graded by their teachers at the age of 16 years. School grade categories were as follows: 4–6 (4.00–6.99); 7–8 (7.00–8.99); 9–10 (9.00–10.00) [27].

#### Health-related risk factors at age 14 years

The same questionnaire from 1980 that was used to measure psychosocial risk factors at age 14 years was used to gather information about subjects’ health behaviour (*alcohol and smoking consumption, frequency of sport hobbies and BMI*).

### Missing data

Since most of the data were acquired through questionnaires, we had a varying level of missing data, from 0 to 32.8%, in different variables with the average proportion of missing data being 9.2% in the PD group. The highest amount of missing data was in the variable *standing without support* (32.8%).

### Statistical analyses

Contingency tables were first applied to analyse frequency distribution of the variables in different diagnostic groups. We used Cox proportional-hazards model to calculate the hazard ratio for each risk factor separately comparing PD to HC. The end point in the Cox regression analysis was illness onset, death, moving abroad or the end of 2016. We did a multivariate Cox regression model in which we chose those variables that had a low *p* value (*p* < 0.1) in one or more variable categories in the univariate analysis comparing PD to HC. If there were several parental mental illness variables with a low *p* value, we chose the one with the lowest *p* value. The multivariate model included four variables (Table 2). For comparisons between different disorder groups, we used Pearson’s Chi-square test or Fisher’s exact test. We used SPSS version 25 for statistical analyses (https://www.ibm.com/analytics/spss-statistics-software).

**Table 2:** Multivariate Cox regression model for risk factors of PD

Variables	HR for PD (95% CI)
Parent’s any psychiatric illness (either parent or both)	
No	Ref
Yes	3.59 (1.84–7.04)
Family type in 1980	
Two-parent family	Ref
Single-parent family or no parents	1.59 (0.79–3.19)
Grade of physical education in 1982	
4–6	1.03 (0.37–2.89)
7–8	Ref
9–10	0.29 (0.11–0.73)
Unwantedness of pregnancy	
Wanted or mistimed	Ref
Unwanted	1.24 (0.55–2.79)

## Results

### Characteristics of the sample

**Fig. 1:**
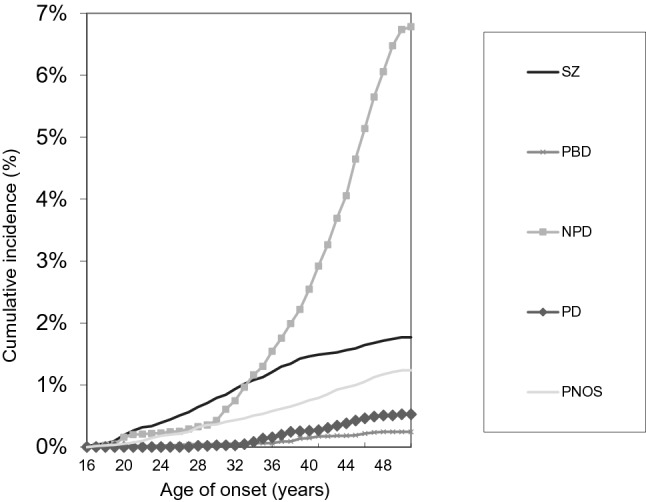
Cumulative incidence of psychotic depression (PD), non-psychotic depression (NPD), schizophrenia (SZ), psychotic bipolar disorder (PBD) and other psychoses (PNOS)

There were 58 subjects with a hierarchical lifetime diagnosis of PD at the end of 2016. Lifetime prevalence of PD was 0.5%. The proportion of females was 60.3%. The comparison groups were as follows: NPD (*n* = 746), SZ (*n* = 195), PBD (*n *= 27), PNOS (*n* = 136) and HC (*n* = 8200). Cumulative incidences of the groups are presented in Fig. 1.

### Univariate analysis of PD risk factors when compared to HC

Compared to HC, PD subjects were significantly more likely to have a parent/parents with any psychiatric illness (HR 3.63 (1.99–6.62)), any psychosis (HR 3.89 (1.41–10.73) and schizophrenia (HR 4.60 (1.44–14.71)), but not a parent with depression or bipolar disorder. Results regarding parental mental illness are presented in the Supplementary Table S1.

Lower frequency of sport hobbies (at most once a fortnight: HR 2.03 (1.00–4.12) at the age of 14 years was a significant risk factor for PD, while a higher school sport grade was a protective factor (HR 0.37 (0.16–0.82)). Another significant finding was that living in a single-parent family or without parents at the age of 14 years increased the risk for the development of PD (HR 2.12 (1.11–4.08). (Supplementary Tables S2 and S4-S5).

Most of the findings remained nonsignificant. None of the psychosocial risk factors during birth showed significant risk. Also, the amount of hospitalizations of the parents of study subjects, due to somatic illness, were few in number (*n* = 3) and the difference nonsignificant. (Supplementary Tables S1–S2).

Birth parameters (birth weight, gestational age, birth weight/gestational age) in the PD group had more deviation from the normal range than in the HC group. There were still no statistically significant differences between the PD and HC groups. Findings regarding motor development and maternal and paternal age were also nonsignificant. (Supplementary Table S3).

Most variables at age 14 years showed no significant risk. School performance in the PD group was worse in all studied variables, but differences did not reach statistical significance. Alcohol and smoking habits showed no distinctive characteristics in comparison to the HC group (Supplementary Tables S2, S4 and S5).

### Multivariate Cox regression model

We included, in the multivariate model, the variables that had a *p* value under 0.1 in one or more categories in the univariate Cox regression analysis when PD was compared to HC. Regarding parental mental illness, there were several variables with *p* < 0.1, and we selected *any parental mental illness*, which had the strongest effect. Out of two variables describing physical activity, we selected *grade of physical education*, also due to the stronger effect. We excluded *mean grade of non-theoretical school subjects*, since it included *grade of physical education.* After this, four variables were included in the multivariate model (Table 2). After adjusting for covariates in the multivariate model, only *any parental mental illness* (HR 3.59 (1.84–7.04)) remained a strong risk factor and high *grade of physical education* (HR 0.29 (0.11–0.73)) a protective factor of PD.

### Comparison to other disorder groups

We compared PD risk factors to all other disorder groups. Psychiatric illness among parents was significantly more common in the PD group when compared to the NPD group (*p* = 0.031). Other group comparisons regarding parental mental illness remained nonsignificant. Otherwise, there were only a few significant differences between disorder groups. The proportion of subjects at lower school level was smaller in PD (5.4%) (*p* = 0.031) than in SZ (17.4%). Also, PD subjects more often (14.6%) (*p* = 0.032) had a deceased sibling than SZ subjects (4.8%).

## Discussion

We analysed several early life risk factors of PD for the first time in a prospective study. The 0.5% prevalence rate of PD in the sample was in line with previous findings [2, 28]. A significant finding in this study was a high prevalence of mental illness in the families of PD subjects, and higher than that of those with NPD. Otherwise, there were only few significant differences between PD and other disorder groups. PD subjects had more often a deceased sibling and were less often at a lower school level than schizophrenia subjects. We also found PD subjects, compared to HC, to have lower sports grades and a lower frequency of sport hobbies in adolescence, and they more often grew up in a single-parent family.

The proportion of psychosis and schizophrenia, but not depression or bipolar disorder, was found to be significantly more common among parents in PD compared to the HC group. Findings concerning parental mental illness in general and psychosis are in line with previous population-representative research [8], but our finding of no bipolar disorder among parents of the study subjects was surprising and conflicting [5]. The fact that in our study non-psychotic depression of the parent was not more common among PD subjects than among any other group, including HC group, is likely due to the small sample size, the unavailability of outpatient diagnoses before 1998 and changing diagnostic practices through the decades (see “Strengths and limitations” section).

Both lower school sports grades and reported frequency of sport hobbies may reflect low physical activity. After adjusting for covariates in the multivariate cox regression model, a higher sports grade remained a significant protective factor for PD compared to HC. Previously, people with major depression are known to have lower levels of physical activity [29] and higher levels of physical activity are known to prevent the development of depression [30]. Also, low levels of physical activity in childhood and adolescence increase the risk of non-affective psychosis later in life, possibly contributed to by deviant motor development [31]. However, we did not find significant differences of motor development in PD, which was consistent with previous research limiting deviant motor development to non-affective psychoses [32]. It is also important to notice that sports grade is influenced by a variety of factors other than physical activity such as physical health, motivation, interpersonal capabilities and genetic factors.

It has been argued that the role of physical activity should be increased in the treatment of depression [33] and severe mental illness in general [34]. Our results support the view that low physical activity is a transdiagnostic marker for later illness across the spectrum of mental illness and a relevant target for prevention and treatment.

Single parenthood has been associated with multiple negative mental health outcomes [35], but many factors associated with single parenthood contribute significantly to this effect [36]. In line with these findings, in our sample, single parenthood or living without parents was a significant risk factor for PD compared to HC in the univariate analysis, but not after adjusting for covariates in the multivariate model.

Our results, with a majority of nonsignificant findings, are also worth considering more closely. It is possible that internal heterogeneity of the diagnostic category of PD partly causes the lack of findings. It is already known that there are significant clinical differences between psychotic depression that has its onset in early adulthood versus psychotic depression in old age [2, 37]. In addition, major depression in general is a highly heterogeneous entity [38]. There might also be significant internal heterogeneity due to different aetiologies in the same age group of PD patients, contributing to the lack of significant risk factors. More long-term follow-up studies focusing on this subject would be likely to have much to offer for the understanding and individualized treatment of PD patients.

### Strengths and limitations

Our data come from a representative birth cohort, with a prospective follow-up from mid-pregnancy 1965–1966 until 2016, approximately 50 years in total. Also, the utilization of national registers gives us a reliable estimate of the treated psychiatric illness both in the cohort subjects and in their families. The method of hierarchical lifetime diagnosis gave us the possibility to examine distinctly those PD patients whose diagnosis did not convert to other psychotic illness by the age of 50 years. A major strength was also that we were able to study many early life variables in the same study. There was some, but not an extensive amount of missing data, the average proportion in the PD group being 9.2%.

The validity of register diagnoses is unfortunately not optimal. However, we believe that the long follow-up (up to 50 years) and our ability to obtain diagnoses since birth for hospitalizations and since 1998 for specialized outpatient care increase this validity significantly. After excluding those subjects who had also been diagnosed with a schizophrenia spectrum disorder, delusional disorder or bipolar disorder during their lifetime, our sample included 58 subjects whose most severe lifetime diagnosis was PD. If the PD diagnosis would have been wrong and the patient was suffering from another severe psychotic disorder, the patient would have likely been diagnosed with that particular disorder later on. The other possibility of misdiagnosis is overdiagnosis, in which the patient was diagnosed with PD when the patient was in fact suffering from non-psychotic depression. The likelihood of this, in our opinion, is low, since the psychotic symptoms are more likely to be missed than overdiagnosed in depression [39].

One limitation in the study was the use of changing diagnostic systems (ICD 8–10) over the decades. We know that the prevalence of affective psychoses has increased in Northern Finland [40], possibly contributed to by a change in diagnostic practices. This especially concerns our finding of only a few cases of depression or bipolar disorder in the parents of PD subjects. Diagnostic practices in Finland have likely shifted to a more prevalent diagnosis of depression [41], whereas the use of other diagnoses was more common during ICD-8 and ICD-9. This may have led to an underestimation of the prevalence of depression or bipolar disorder among the parents of PD patients.

The sample size of PBD was unfortunately low (*n* = 27), reducing the reliability of the results concerning this diagnostic group. However, we wanted to include this important comparison group since this is the first prospective study to examine the early risk factors of PD. Also, PBD has not been extensively studied separately from non-psychotic bipolar disorder.

Also, the unavailable information of outpatient care diagnoses before the year 1998 means that some early-onset cases with milder symptoms are likely to have been missed. This is not a concern in the PD group with a very high hospitalization rate [20], but it is likely that in our study the NPD group represents a more severe portion of illness cases.

Because of lack of data, we were not able to analyse the effects of certain potential risk factors. Cannabis use, migration and the majority of childhood traumatic events are examples of such risk factors that were not possible to study here. In addition, we studied only risk factors in early childhood and adolescence, while many factors later in life may increase the risk of PD.

Our study also has a limitation regarding the age of the sample, since we were not able to include subjects who were diagnosed with PD after the age of 50 years. Therefore, our results should not be interpreted to be representative of late-onset PD, for which other factors might cause predispositions.

We consider our sample size of 58 in the PD group to be relatively small, but sufficient to discover potential robust risk factors. However, it is still possible that smaller effects may have gone unnoticed. Altogether, our findings in this explorative study are to be regarded as preliminary, as no comprehensive adjustment for confounding variables was performed. Also, we did multiple comparisons, which means that chance findings are possible.

## Conclusions

Low school sports grade in adolescence and familial mental illness were common factors in PD in our study. The PD risk factor profile was similar to that of other mental illness in most variables. A small number of significant risk factors may point to underlying heterogeneity. Research on the risk factors of PD should also be carried out in other samples to reduce the gap in knowledge.

## Electronic supplementary material

Below is the link to the electronic supplementary material.

Supplementary file1 (DOCX 54 kb)
