# Supplementary material for: Early childhood and adolescent risk factors for psychotic depression in a general population birth cohort sample
Source: Soc Psychiatry Psychiatr Epidemiol. 2020 Feb 13;55(9):1179–86. doi: 10.1007/s00127-020-01835-7 (PMC7471190; doi:10.1007/s00127-020-01835-7)
Supplement: Supplementary file 1 — Supplementary file1 (DOCX 54 kb) [file 127_2020_1835_MOESM1_ESM.docx]

**Early childhood and adolescent risk factors for psychotic depression in a general population birth cohort sample**

Miika Nietola¹, Hanna Huovinen, Anni Heiskala, Tanja Nordström, Jouko Miettunen, Jyrki Korkeila, Erika Jääskeläinen

¹ Psychiatric department, University of Turku and the Hospital District of Southwest Finland, Finland. Corresponding author; [mtniet@utu.fi](mailto:mtniet@utu.fi)

Journal: Social Psychiatry and Psychiatric Epidemiology

**Supplement Table S1. Psychiatric and somatic illness of the parents**

| **Variables** | Psychotic Depression  (PD) (n=58) | | Non-psychotic Depression  (NPD) (n=746) | | Schizophrenia  (SZ)  (n =195) | | Psychotic Bipolar  Disorder (PBD)  (n = 27) | | Other  Psychoses (PNOS)  (n=136) | | Healthy Controls (HC)  (n=8200) | | HR for PD (95% CI) |
| --- | --- | --- | --- | --- | --- | --- | --- | --- | --- | --- | --- | --- | --- |
|  | n | % | n | % | n | % |  |  | n | % | n | % |  |
| **Father’s somatic hospitalization (≥30days) before 1982** |  |  |  |  |  |  |  |  |  |  |  |  |  |
| no | 49 | 96.1% | 642 | 90.7% | 154 | 93.9% | 24 | 92.3% | 109 | 93.2% | 7285 | 94.1% |  |
| yes | 2 | 3.9% | 66 | 9.3% | 10 | 6.1% | 2 | 7.7% | 8 | 6.8% | 459 | 5.9% | 0.65 (0.16-2.66) |
| **Mother’s somatic hospitalization (≥30days) before 1982** |  |  |  |  |  |  |  |  |  |  |  |  |  |
| no | 50 | 98.0% | 671 | 94.8% | 154 | 93.9% | 24 | 92.3% | 107 | 91.5% | 7345 | 94.8% |  |
| yes | 1 | 2.0% | 37 | 5.2% | 10 | 6.1% | 2 | 7.7% | 10 | 8.5% | 399 | 5.2% | 0.37 (0.05-2.67) |
| **Parent’s any psychiatric illness (either parent or both)** |  |  |  |  |  |  |  |  |  |  |  |  |  |
| no | 44 | 75.9% | 646 | 86.6% | 154 | 79.0% | 20 | 74.1% | 109 | 80.1% | 7537 | 91.9% |  |
| yes | 14 | 24.1% | 100 | 13.4% | 41 | 21.0% | 7 | 25.9% | 27 | 19.9% | 663 | 8.1% | 3.63* (1.99-6.62) |
| **Parent’s depression (either parent or both)** |  |  |  |  |  |  |  |  |  |  |  |  |  |
| no | 56 | 96.6% | 711 | 95.3% | 184 | 94.4% | 24 | 88.9% | 124 | 91.2% | 8041 | 98.1% |  |
| yes | 2 | 3.4% | 35 | 4.7% | 11 | 5.6% | 3 | 11.1% | 12 | 8.8% | 159 | 1.9% | 1.81 (0.44-7.43) |
| **Parent’s psychosis (either parent or both)** |  |  |  |  |  |  |  |  |  |  |  |  |  |
| no | 54 | 93.1% | 720 | 96.5% | 181 | 92.8% | 22 | 81.5% | 130 | 95.6% | 8048 | 98.1% |  |
| yes | 4 | 6.9% | 26 | 3.5% | 14 | 7.2% | 5 | 18.5% | 6 | 4.4% | 152 | 1.9% | 3.89 (1.41-10.73) |
| **Parent’s any bipolar disorder (either parent or both)** |  |  |  |  |  |  |  |  |  |  |  |  |  |
| no | 58 | 100.0% | 745 | 99.9% | 195 | 100.0% | 25 | 92.6% | 135 | 99.3% | 8188 | 99.9% |  |
| yes | 0 | 0.0% | 1 | 0.1% | 0 | 0.0% | 2 | 7.4% | 1 | 0.7% | 12 | 0.1% | incalculable |
| **Parent’s schizophrenia (either parent or both)** |  |  |  |  |  |  |  |  |  |  |  |  |  |
| no | 55 | 94.8% | 732 | 98.1% | 185 | 94.9% | 25 | 92.6% | 132 | 97.1% | 8106 | 98.9% |  |
| yes | 3 | 5.2% | 14 | 1.9% | 10 | 5.1% | 2 | 7.4% | 4 | 2.9% | 94 | 1.1% | 4.60 (1.44-14.71) |
| **Parent’s any alcohol use disorder (either parent or both)** |  |  |  |  |  |  |  |  |  |  |  |  |  |
| no | 54 | 93.1% | 712 | 95.4% | 185 | 94.9% | 25 | 92.6% | 128 | 94.1% | 7978 | 97.3% |  |
| yes | 4 | 6.9% | 34 | 4.6% | 10 | 5.1% | 2 | 7.4% | 8 | 5.9% | 222 | 2.7% | 2.71 (0.98-7.47) |

(* = PD vs. NPD. chi-square -test. p=0.031)

**Supplement Table S2. Psychosocial risk factors**

| **Variables** | Psychotic Depression  (PD) (n=58) | | Non-psychotic Depression  (NPD) (n=746) | | Schizophrenia  (SZ)  (n =195) | | Psychotic Bipolar  Disorder (PBD)  (n = 27) | | Other  Psychoses (PNOS)  (n=136) | | Healthy Controls (HC)  (n=8200) | | HR for PD (95% CI) |
| --- | --- | --- | --- | --- | --- | --- | --- | --- | --- | --- | --- | --- | --- |
| ***Risk factors during birth*** |  |  |  |  |  |  |  |  |  |  |  |  |  |
| **Unwantedness of pregnancy** |  |  |  |  |  |  |  |  |  |  |  |  |  |
| wanted or mistimed | 45 | 81.8% | 566 | 87.9% | 129 | 79.6% | 22 | 100.0% | 100 | 87.0% | 6337 | 88.8% |  |
| unwanted | 10 | 18.2% | 78 | 12.1% | 33 | 20.4% | 0 | 0.0% | 15 | 13.0% | 796 | 11.2% | 1.79 (0.90-3.55) |
| **Multiparity** |  |  |  |  |  |  |  |  |  |  |  |  |  |
| no (1-5 earlier births) | 49 | 84.5% | 654 | 87.9% | 160 | 82.5% | 24 | 92.3% | 113 | 83.1% | 7250 | 88.6% |  |
| yes (≥6 earlier births) | 9 | 15.5% | 90 | 12.1% | 34 | 17.5% | 2 | 7.7% | 23 | 16.9% | 937 | 11.4% | 1.43 (0.70-2.91) |
| **Urbanicity** |  |  |  |  |  |  |  |  |  |  |  |  |  |
| rural | 41 | 70.7% | 535 | 71.7% | 140 | 71.8% | 21 | 77.8% | 102 | 75.0% | 6003 | 73.2% |  |
| urban | 17 | 29.3% | 211 | 28.3% | 55 | 28.2% | 6 | 22.2% | 34 | 25.0% | 2197 | 26.8% | 1.14 (0.65-2.00) |
| **Social class 1966** |  |  |  |  |  |  |  |  |  |  |  |  |  |
| unskilled | 10 | 18.5% | 159 | 22.8% | 53 | 28.6% | 7 | 26.9% | 36 | 28.1% | 1689 | 21.5% |  |
| others | 44 | 81.5% | 538 | 77.2% | 132 | 71.4% | 19 | 73.1% | 92 | 71.9% | 6183 | 78.5% | 1.19 (0.60-2.36) |
| **Maternal education** |  |  |  |  |  |  |  |  |  |  |  |  |  |
| low | 8 | 15.1% | 55 | 8.5% | 10 | 6.3% | 0 | 0.0% | 8 | 6.8% | 658 | 9.2% | 1.60 (0.73-3.47) |
| interm. | 31 | 58.5% | 393 | 60.6% | 92 | 57.5% | 17 | 77.3% | 73 | 62.4% | 4043 | 56.3% |  |
| high | 14 | 26.4% | 200 | 30.9% | 58 | 36.3% | 5 | 22.7% | 36 | 30.8% | 2477 | 34.5% | 0.74 (0.39-1.39) |
| **Maternal depression during pregancy** |  |  |  |  |  |  |  |  |  |  |  |  |  |
| no depression | 44 | 81.5% | 543 | 83.4% | 131 | 80.9% | 19 | 86.4% | 92 | 82% | 6206 | 86.9% |  |
| depressed or very depressed | 10 | 18.5% | 108 | 16.6% | 31 | 19.1% | 3 | 13.6% | 23 | 20% | 934 | 13.1% | 1.52 (0.76-3.02) |
|  |  |  |  |  |  |  |  |  |  |  |  |  |  |
| ***Risk factors at age 14*** |  |  |  |  |  |  |  |  |  |  |  |  |  |
| **Deceased siblings** |  |  |  |  |  |  |  |  |  |  |  |  |  |
| No | 41 | 85.4% | 552 | 89.2% | 138 | 95.2% | 20 | 95.2% | 86 | 85.1% | 6273 | 90.6% |  |
| Yes | 7 | 14.6% | 67 | 10.8% | 7 | 4.8% | 1 | 4.8% | 15 | 14.9% | 649 | 9.4% | 1.68* (0.75-3.74) |
| **Family type in 1980** |  |  |  |  |  |  |  |  |  |  |  |  |  |
| Two-parent family | 36 | 75.0% | 495 | 80.1% | 117 | 80.1% | 18 | 85.7% | 85 | 85.0% | 5976 | 86.3% |  |
| Single-parent family or no parents | 12 | 25.0% | 123 | 19.9% | 29 | 19.9% | 3 | 14.3% | 15 | 15.0% | 952 | 13.7% | 2.12 (1.11-4.08) |
| **Social class 1980** |  |  |  |  |  |  |  |  |  |  |  |  |  |
| higher | 12 | 24.0% | 199 | 29.0% | 53 | 30.5% | 8 | 33.3% | 30 | 26.3% | 2430 | 31.4% |  |
| lower | 30 | 60.0% | 406 | 59.2% | 103 | 59.2% | 14 | 58.3% | 65 | 57.0% | 4292 | 55.5% | 1.42 (0.73-2.77) |
| other | 8 | 16.0% | 81 | 11.8% | 18 | 10.3% | 2 | 8.3% | 19 | 16.7% | 1010 | 13.1% | 1.57 (0.64-3.84) |
| **Moving home town in 1966–1982** |  |  |  |  |  |  |  |  |  |  |  |  |  |
| No | 44 | 86.3% | 616 | 87.4% | 141 | 86.5% | 24 | 96.0% | 101 | 86.3% | 6665 | 86.3% |  |
| 1-2 times | 7 | 13.7% | 89 | 12.6% | 22 | 13.5% | 1 | 4.0% | 16 | 13.7% | 1061 | 13.7% | 1.02 (0.46-2.26) |
| **Mother’s work** |  |  |  |  |  |  |  |  |  |  |  |  |  |
| At home | 25 | 53.2% | 269 | 44.8% | 79 | 54.9% | 13 | 61.9% | 49 | 49.5% | 3017 | 44.4% |  |
| At work | 22 | 46.8% | 332 | 55.2% | 65 | 45.1% | 8 | 38.1% | 50 | 50.5% | 3782 | 55.6% | 0.71 (0.40-1.26) |

(* = PD vs. SZ. Fisher’s exact test. p=0.047)

**Supplement Table S3. Biological risk factors**

| **Variables** | Psychotic Depression  (PD) (n=58) | | Non-psychotic Depression  (NPD) (n=746) | | Schizophrenia  (SZ)  (n =195) | | Psychotic Bipolar  Disorder (PBD)  (n = 27) | | Other  Psychoses (PNOS)  (n=136) | | Healthy Controls (HC)  (n=8200) | | HR for PD (95% CI) |
| --- | --- | --- | --- | --- | --- | --- | --- | --- | --- | --- | --- | --- | --- |
|  | n | % | n | % | n | % |  |  | n | % | n | % |  |
| **Gender** |  |  |  |  |  |  |  |  |  |  |  |  |  |
| male | 23 | 39.7% | 341 | 45.8% | 114 | 58.5% | 11 | 40.7% | 79 | 58.1% | 4126 | 50.3% |  |
| female | 35 | 60.3% | 403 | 54.2% | 81 | 41.5% | 16 | 59.3% | 57 | 41.9% | 4072 | 49.7% | 1.53 (0.90-2.58) |
| **Birth weight** |  |  |  |  |  |  |  |  |  |  |  |  |  |
| <2500g | 4 | 6.9% | 30 | 4.0% | 11 | 5.6% | 0 | 0.0% | 5 | 3.7% | 278 | 3.4% | 2.20 (0.80-6.09) |
| 2500-4500g | 51 | 87.9% | 693 | 92.9% | 174 | 89.2% | 27 | 100.0% | 128 | 94.1% | 7694 | 93.8% |  |
| >4500g | 3 | 5.2% | 23 | 3.1% | 10 | 5.1% | 0 | 0.0% | 3 | 2.2% | 227 | 2.8% | 1.97 (0.62-6.32) |
| **Gestational age** |  |  |  |  |  |  |  |  |  |  |  |  |  |
| ≤37 weeks | 5 | 10.0% | 34 | 5.4% | 9 | 5.7% | 0 | 0.0% | 7 | 6.3% | 338 | 4.8% | 2.15 (0.84-5.47) |
| 37-42 weeks | 37 | 74.0% | 477 | 75.8% | 123 | 78.3% | 15 | 68.2% | 87 | 77.7% | 5355 | 75.9% |  |
| ≥42 weeks | 8 | 16.0% | 118 | 18.8% | 25 | 15.9% | 7 | 31.8% | 18 | 16.1% | 1366 | 19.4% | 0.85 (0.40-1.83) |
| **Birthweight/**  **Gestational age** |  |  |  |  |  |  |  |  |  |  |  |  |  |
| ≤ -2SD | 2 | 4.0% | 20 | 3.2% | 9 | 5.7% | 0 | 0.0% | 2 | 1.8% | 165 | 2.3% | 1.81 (0.44-7.45) |
| -2SD-+2SD | 45 | 90.0% | 584 | 92.8% | 141 | 89.8% | 22 | 100.0% | 110 | 98.2% | 6728 | 95.3% |  |
| ≥ +2SD | 3 | 6.0% | 25 | 4.0% | 7 | 4.5% | 0 | 0.0% | 0 | 0.0% | 166 | 2.4% | 2.64 (0.82-8.49) |
| **Perinatal problem** |  |  |  |  |  |  |  |  |  |  |  |  |  |
| No | 50 | 86.2% | 667 | 89.4% | 175 | 89.7% | 26 | 96.3% | 120 | 88.2% | 7443 | 90.8% |  |
| Yes | 8 | 13.8% | 79 | 10.6% | 20 | 10.3% | 1 | 3.7% | 16 | 11.8% | 757 | 9.2% | 1.60 (0.76-3.37) |
| **Maternal smoking** |  |  |  |  |  |  |  |  |  |  |  |  |  |
| sustained | 8 | 14.8% | 112 | 17.3% | 17 | 10.7% | 7 | 31.8% | 25 | 21.9% | 997 | 14.0% | 1.15 (0.54-2.47) |
| stopped | 6 | 11.1% | 42 | 6.5% | 8 | 5.0% | 1 | 4.5% | 6 | 5.3% | 469 | 6.6% | 1.83 (0.78-4.32) |
| no smoking | 40 | 74.1% | 495 | 76.3% | 134 | 84.3% | 14 | 63.6% | 83 | 72.8% | 5672 | 79.5% |  |
| **Maternal age** |  |  |  |  |  |  |  |  |  |  |  |  |  |
| ≤19 years | 4 | 6.9% | 79 | 10.6% | 16 | 8.2% | 1 | 3.7% | 14 | 10.3% | 791 | 9.6% | 0.68 (0.25-1.89) |
| 20-35 years | 47 | 81.0% | 567 | 76.0% | 145 | 74.4% | 24 | 88.9% | 101 | 74.3% | 6264 | 76.4% |  |
| ≥35 years | 7 | 12.1% | 100 | 13.4% | 34 | 17.4% | 2 | 7.4% | 21 | 15.4% | 1145 | 14.0% | 0.82 (0.37-1.81) |
| **Paternal age** |  |  |  |  |  |  |  |  |  |  |  |  |  |
| ≤19 years | 8 | 15.4% | 158 | 22.5% | 36 | 20.0% | 7 | 28.0% | 35 | 28.0% | 1709 | 22.1% | 0.65 (0.31-1.40) |
| 20-35 years | 38 | 73.1% | 467 | 66.5% | 123 | 68.3% | 16 | 64.0% | 74 | 59.2% | 5274 | 68.2% |  |
| ≥35 years | 6 | 11.5% | 77 | 11.0% | 21 | 11.7% | 2 | 8.0% | 16 | 12.8% | 752 | 9.7% | 1.12 (0.47-2.64) |
| **Walking without support** |  |  |  |  |  |  |  |  |  |  |  |  |  |
| <11 months | 8 | 19% | 118 | 21.1% | 22 | 17.6% | 6 | 31.6% | 18 | 19.6% | 1493 | 24.1% | 0.80 (0.36-1.75) |
| 11-13 months | 28 | 66.7% | 375 | 67.2% | 76 | 60.8% | 10 | 52.6% | 59 | 64.1% | 4139 | 66.9% |  |
| ≥14 months | 6 | 14.3% | 65 | 11.6% | 27 | 21.6% | 3 | 15.8% | 15 | 16.3% | 554 | 9.0% | 1.60 (0.66—3.86) |
| **Standing without support** |  |  |  |  |  |  |  |  |  |  |  |  |  |
| <10 months | 8 | 20.5% | 115 | 20.3% | 21 | 14.6% | 6 | 30.0% | 19 | 19.2% | 1585 | 24.2% | 0.84 (0.38-1.88) |
| 10-11 months | 23 | 59% | 348 | 61.5% | 79 | 54.9% | 9 | 45% | 52 | 52.5% | 3790 | 57.8% |  |
| ≥12 months | 8 | 20.5% | 103 | 18.2% | 44 | 30.6% | 5 | 25% | 28 | 28.3% | 1181 | 18.0% | 1.12 (0.50-2.50) |

**Supplement Table S4.** **School performance and risk of psychotic depression and other mental disorders**

| **Variables** | Psychotic Depression  (PD) (n=58) | | Non-psychotic Depression  (NPD) (n=746) | | Schizophrenia  (SZ)  (n =195) | | Psychotic Bipolar  Disorder (PBD)  (n = 27) | | Other  Psychoses (PNOS)  (n=136) | | Healthy Controls (HC)  (n=8200) | | HR for PD (95% CI) |
| --- | --- | --- | --- | --- | --- | --- | --- | --- | --- | --- | --- | --- | --- |
|  | n | % | n | % | n | % |  |  | n | % | n | % |  |
| **Mean grade of theoretical school subjects** |  |  |  |  |  |  |  |  |  |  |  |  |  |
| 4-6 | 22 | 43.1% | 296 | 42.4% | 67 | 42.1% | 8 | 32.0% | 58 | 51.3% | 2704 | 35.4% | 1.29 (0.73-2.26) |
| 7-8 | 27 | 52.9% | 359 | 51.4% | 78 | 49.1% | 16 | 64.0% | 47 | 41.6% | 4188 | 54.8% |  |
| 9-10 | 2 | 3.9% | 43 | 6.2% | 14 | 8.8% | 1 | 4.0% | 8 | 7.1% | 744 | 9.7% | 0.42 (0.10-1.77) |
| **Mean grade of non-theoretical school subjects** |  |  |  |  |  |  |  |  |  |  |  |  |  |
| 4-6 | 7 | 13.7% | 78 | 11.2% | 25 | 15.7% | 2 | 8.0% | 22 | 19.5% | 528 | 6.9% | 2.17 (0.97-4.83 |
| 7-8 | 41 | 80.4% | 577 | 82.7% | 124 | 78.0% | 20 | 80.0% | 86 | 76.1% | 6417 | 84.0% |  |
| 9-10 | 3 | 5.9% | 43 | 6.2% | 10 | 6.3% | 3 | 12.0% | 5 | 4.4% | 691 | 9.0% | 0.68 (0.21-2.19) |
| **Grade of physical education** |  |  |  |  |  |  |  |  |  |  |  |  |  |
| 4-6 | 6 | 11.5% | 93 | 13.0% | 22 | 11.9% | 2 | 7.7% | 17 | 13.3% | 525 | 6.6% | 1.49 (0.63-3.52) |
| 7-8 | 39 | 75.0% | 470 | 65.7% | 125 | 67.6% | 17 | 65.4% | 77 | 60.2% | 4979 | 62.7% |  |
| 9-10 | 7 | 13.5% | 152 | 21.3% | 38 | 20.5% | 7 | 26.9% | 34 | 26.6% | 2442 | 30.7% | 0.37 (0.16-0.82) |
| **Mean grade of all school subjects** |  |  |  |  |  |  |  |  |  |  |  |  |  |
| 4-6 | 19 | 37.3% | 245 | 35.1% | 56 | 35.2% | 6 | 24.0% | 52 | 46.0% | 2091 | 27.4% | 1.58 (0.89-2.82) |
| 7-8 | 29 | 56.9% | 416 | 59.6% | 91 | 57.2% | 18 | 72.0% | 55 | 48.7% | 4950 | 64.8% |  |
| 9-10 | 3 | 5.9% | 37 | 5.3% | 12 | 7.5% | 1 | 4.0% | 6 | 5.3% | 595 | 7.8% | 0.86 (0.26-2.84) |
| **School level in 1980** |  |  |  |  |  |  |  |  |  |  |  |  |  |
| Normal or upper class | 53 | 94.6% | 683 | 92.9% | 161 | 82.6% | 26 | 96.3% | 115 | 85.2% | 7737 | 95.4% |  |
| Below normal class or customised school | 3 | 5.4% | 52 | 7.1% | 34 | 17.4% | 1 | 3.7% | 20 | 14.8% | 377 | 4.6% | 1.25* (0.39-4.01) |

(* = PD vs. SZ. chi-square -test and Fisher’s exact test. p=0.031)

**Supplement Table S5. Health behavior related risk factors at age 14**

| **Variables** | Psychotic Depression  (PD) (n=58) | | Non-psychotic Depression  (NPD) (n=746) | | Schizophrenia  (SZ)  (n =195) | | Psychotic Bipolar  Disorder (PBD)  (n = 27) | | Other  Psychoses (PNOS)  (n=136) | | Healthy Controls (HC)  (n=8200) | | HR for PD (95% CI) |
| --- | --- | --- | --- | --- | --- | --- | --- | --- | --- | --- | --- | --- | --- |
|  | n | % | n | % | n | % |  |  | n | % | n | % |  |
| **Alcohol use at the age of 14** |  |  |  |  |  |  |  |  |  |  |  |  |  |
| Had never drunk alcohol | 21 | 45.7% | 237 | 38.5% | 54 | 37.5% | 7 | 33.3% | 44 | 44.4% | 2870 | 41.6% |  |
| Had drunk alcohol once or few times | 25 | 54.3% | 359 | 58.3% | 88 | 61.1% | 11 | 52.4% | 54 | 54.5% | 3866 | 56.0% | 0.89 (0.50-1.58) |
| Occasional or regular use of alcohol | 0 | 0.0% | 20 | 3.2% | 2 | 1.4% | 3 | 14.3% | 1 | 1.0% | 163 | 2.4% | incalculable |
| **Smoking at the age of 14** |  |  |  |  |  |  |  |  |  |  |  |  |  |
| Had never smoked | 16 | 34.0% | 174 | 28.2% | 49 | 34.0% | 4 | 19.0% | 29 | 29.0% | 2311 | 33.5% |  |
| Had smoked once or few times | 21 | 44.7% | 284 | 46.1% | 74 | 51.4% | 9 | 42.9% | 47 | 47.0% | 3501 | 50.7% | 0.87 (0.45-1.67) |
| Occasional or regular smoking | 10 | 21.3% | 158 | 25.6% | 21 | 14.6% | 8 | 38.1% | 24 | 24.0% | 1092 | 15.8% | 1.35 (0.61-2.97) |
| **Frequency of sport hobbies at the age of 14** |  |  |  |  |  |  |  |  |  |  |  |  |  |
| At least every second day | 14 | 30.4% | 215 | 35.0% | 52 | 36.6% | 8 | 38.1% | 29 | 29.3% | 2652 | 38.9% |  |
| At least once a week | 15 | 32.6% | 229 | 37.3% | 48 | 33.8% | 6 | 28.6% | 39 | 39.4% | 2589 | 38.0% | 1.09 (0.53-2.26) |
| At most once a fortnight | 17 | 37.0% | 170 | 27.7% | 42 | 29.6% | 7 | 33.3% | 31 | 31.3% | 1572 | 23.1% | 2.03 (1.00-4.12) |
| **BMI at the age of 14** |  |  |  |  |  |  |  |  |  |  |  |  |  |
| ≤17.7 kg/m2 (lowest 25th percentile) | 9 | 17.3% | 161 | 24.1% | 52 | 31.5% | 4 | 18.2% | 35 | 30.4% | 1758 | 23.6% | 0.66 (0.31-1.39) |
| 17.8–20.49 kg/m2 | 30 | 57.7% | 329 | 49.3% | 79 | 47.9% | 14 | 63.6% | 51 | 44.3% | 3888 | 52.2% |  |
| ≥20.5 kg/m2 (highest 25th percentile) | 13 | 25.0% | 177 | 26.5% | 34 | 20.6% | 4 | 18.2% | 29 | 25.2% | 1801 | 24.2% | 0.93 (0.48-1.77) |
